# Maternal mental health and child nutritional status in an urban slum in Bangladesh: A cross-sectional study

**DOI:** 10.1371/journal.pgph.0000871

**Published:** 2022-10-19

**Authors:** Ahad Mahmud Khan

**Affiliations:** 1 Projahnmo Research Foundation, Dhaka, Bangladesh; 2 Usher Institute, College of Medicine and Veterinary Medicine, University of Edinburgh, Edinburgh, United Kingdom; African Population and Health Research Center, KENYA

## Abstract

Poor mental health may diminish a mother’s capacity to adequately care for her child, resulting in a negative impact on the child’s nutrition. This study aims to determine the association between maternal mental health and child nutritional status in a poor urban population in Bangladesh. We carried out a cross-sectional study among 264 mother-child pairs in an urban slum area of Bangladesh. The Self-Reporting Questionnaire-20 (SRQ-20) was used to assess maternal mental health. An SRQ-20 score ≥7 was considered a common mental disorder (CMD). Anthropometric measurements were performed to assess nutritional status of the children. The prevalence of maternal CMD was 46.2%. Maternal CMD was associated with poorer child feeding practice (p<0.001), poorer hygiene practice (p<0.001), poorer preventive care service use (p = 0.016), and suffering from diarrheal diseases (p = 0.049). The prevalence of stunting, wasting and underweight in children was 44.3%, 18.2% and 33.7%, respectively. A poorer child feeding practice was associated with wasting (p = 0.004) and underweight (p<0.001) but not with stunting. Poorer hygiene practices and suffering from diarrheal diseases were associated with stunting and underweight, but not with wasting. In multivariable analysis, maternal CMD was associated with child wasting (adjusted odds ratio, aOR = 2.25, 95% CI = 1.15–4.43). The association between maternal CMD and child underweight found in the bivariate analysis was attenuated and no longer statistically significant after multivariable analysis (aOR = 1.77, 95% CI = 0.94–3.33). No statistically significant association was observed between maternal CMD and stunting in this study (aOR = 1.46, 95% CI = 0.84–2.54). Maternal mental health affects nutritional status of the children where child feeding practice, hygiene practice and preventive care use might play a role. Interventions to address maternal mental health in child nutrition programs might improve child nutritional status.

## Introduction

Malnutrition is a leading cause of child mortality, morbidity and lifelong developmental impairment [1]. Globally, 162 million under-five children are stunted; 99 million are underweight, and one million are wasted; a major portion of these children reside in Asia [2]. Although the trends in malnutrition prevalence have continued to decrease in recent years in Bangladesh, it is still very high [3]. According to the Bangladesh Demographic and Health Survey (BDHS) 2011, the prevalence of stunting, wasting and underweight was 41%, 16% and 36%, respectively [4]. The contributing factors of child malnutrition include child age, sex, birth weight, previous birth interval, parent’s education, maternal nutrition, low socioeconomic status, child feeding practice, hygiene, child illness, health seeking behavior, etc. [5–7].

Maternal mental health plays a major contributing role in child nutritional status [8–12]. Common mental disorders (CMDs) such as anxiety, depression, and somatic symptoms [13] are more frequent among women than men [14]. CMDs are fairly common among mothers of under-five children, especially in developing countries [10, 11]. In a multi-country study, the prevalence of maternal CMDs ranged from 21% in Vietnam to 33% in Peru [10]. In Bangladesh, the prevalence has been reported to be 49% [11].

When the mother’s mental health is compromised, it adversely affects child nutrition [8–12], interfering with a mother’s ability to care for her child [15]. Maternal CMD affects their caregiving practices, leading to improper feeding and inadequate health care for children, especially immunization [10, 16]. Because of maternal CMD, hygiene practice is not adequately maintained, resulting in child illness, especially diarrhea [11]. There is a bidirectional relationship between infection and malnutrition. The infection causes malnutrition by reducing dietary intake and intestinal absorption, whereas malnutrition increases the risk of diarrhea as it can predispose to infection due to a weak immune system [17]. Insufficient childcare doubles the probability of child malnutrition [18]. The relationship linking maternal mental health to child nutrition is shown in S1 Fig.

Several studies have been published on the association between maternal CMD and child nutritional status [8, 10–12, 19, 20]. However, there is a scarcity of literature focusing on the poor urban community where the prevalence of maternal CMD is elevated [21]. Therefore, this study was designed to assess the relationship between maternal mental health and child nutritional status in the urban slum areas of Bangladesh. This result would help understand the relationship between maternal mental health and child nutrition among the urban population and strengthen the knowledge base of policymakers in decision making to combat child malnutrition.

## Materials and methods

### Study design and setting

The present study was a community-based cross-sectional study carried out from September to November 2013. It was conducted in an urban slum area at Kamrangichar in the Dhaka district. Dhaka is the capital of Bangladesh. It is at the center of national government, trade and culture. Kamrangichar is situated in the southwest part of Dhaka. An estimated 400,000 population lived in its surface area of 3.68 km^2^. The study area is divided into 12 administrative areas or ‘*mohallas’*. This place was selected for this study as it represented a typical urban area.

### Study population and sample size

The study population included mothers and their children aged below five years. Pregnant mothers were excluded because pregnancy has a potential effect on the mother’s mental health [22] as well as on her body mass index (BMI) [23]. Data collection was done from a sample of 264 mother-child pairs. The sample size achieved 43% power at a 10% significance level in detecting the association between maternal CMD and child stunting, based on a study conducted in Bangladesh, where the prevalence of CMD was 44% among the mothers having children suffering from stunting with an odds ratio (OR) of 1.21 [11]. An equal number of participants were recruited from each ‘*mohalla’* of the study area to achieve the sample size. Starting from the center of the ‘*mohalla’*, the data collectors approached a randomly selected direction. They knocked on doors of consecutive households, and if both mother and child were present at that period, they were recruited. In case of multiple under-five children of a mother, the youngest one was enrolled.

### Data collection technique

Data were collected through face-to-face interviews, measurements of height and weight and observations of the cleanliness of the child and mother. The interview was conducted at the participant’s home, ensuring privacy and confidentiality. Informed written consent was obtained before the interview. Anthropometric measurements were performed following standard procedures [24]. Child weight was measured using electronic scales accurate to 100 g. The supine length was taken up to the age of 24 months, and standing height was taken after 24 months using locally manufactured length/height boards that were precise to 1 mm. The height and weight of the mothers were also measured. Their hands, clothes, face and hair were observed for cleanliness. Data were collected from all the participants by a single team of data collectors. Data collectors were hired from the local community, and they were trained by the Principal Investigator.

### Measurements

A structured questionnaire was used that included questions on sociodemographic information, childcare practices and child illness. There were also questions from Self-Reporting Questionnaire-20 (SRQ-20) [25], Household Food Insecurity Access Scale (HFIAS) [26], and Kuppuswamy’s socioeconomic status scale [27]. A checklist for height, weight and hygiene was used. The dependent variables were child nutritional status, i.e., stunting, wasting and underweight. The key independent variables were maternal mental health status and the background characteristics of the sample. According to the study objectives, the variables were identified, and an English questionnaire was drafted. It was validated by an expert who provided feedback on different items. Then the questionnaire was translated into the local language (Bangla) and adapted for the local context discussing with local people. To test the clarity and comprehensibility of contents, the questionnaire was pretested on six respondents of similar background. After necessary modifications, the questionnaire was finalized.

The SRQ-20 developed by the World Health Organization (WHO) was used to measure maternal mental health. It is a 20-item tool that includes questions about depressive, anxiety, panic and somatic symptoms in the preceding four weeks. Each of the 20 items is scored 1 or 0, indicating the presence or absence of symptoms, respectively. The sum of scores generates an overall SRQ-20 scale ranging from 0 to 20, where higher scores indicate poor mental health states and vice versa [25]. A cut-off of 7 was set to categorize women with ‘CMD’ or ‘no CMD’, as suggested by several studies [10, 11, 19, 28]. The reliability and validity of this instrument are well established [25], and it has been used in several studies, including in Bangladesh [10, 11, 19, 28].

The HFIAS 9-item questionnaire measured household food security appropriate for the urban population. The questionnaire asks about a specific condition related to the experience of food insecurity for a period of four weeks preceding the survey. According to their HFIAS questionnaire scores, households were categorized into four groups: food secure, mildly, moderately and severely food insecure [26]. It has been used in different surveys, including in Bangladesh, and the reliability and validity are well established [11, 26].

The original version of Kuppuswamy’s socioeconomic scale (SES) developed in 1976 in India [29] was revised by Kumar et al. in 2012. It is an important tool for measuring SES in urban communities. It consists of three variables: the family head’s education, occupation and monthly family income. SES was categorized as lower, lower-middle, and upper-middle [27].

Family members were relatives living in the same household and shared the same kitchen. The family size was categorized into small (≤4 members), medium (5–6 members) and large (≥7 members), depending on family members. The families were of two types: (i) nuclear family consisting of husband, wife and their children; and (ii) extended family—where more than one nuclear family live together and shares the household functions and income [30].

The maternal occupation was categorized as housewives and working mothers. The women were considered working mothers if they worked outside the home for income, in addition to the work they performed at home. The mothers were considered housewives if they were not involved in any income-generating activity outside the home.

Child feeding practice was measured using the age-specific child feeding index for 0–6, 6–9, 9–12, 12–36 and 36–60 months age groups. The variables used in the index creation were breastfeeding, use of baby feeding in the previous 24 hours, dietary diversity, food group frequency and meal frequency. The general scoring system was to assign a score of -1 for a potentially harmful practice, a score of 0 for medium practice and a score of 1 for positive practice. The final child feeding index was the sum of the scores obtained for each variable [31]. Feeding terciles were calculated to categorize feeding practices into poor, average, and good to make them comparable across age groups.

The child’s and mother’s hygiene was assessed based on a hygiene spot check of the general appearance of hands, clothes, face and hair [32]. Each observation was scored 0 or 1, indicating dirty and clean, respectively. The sum of scores generated an overall hygiene scale (range: 0–4) where higher scores indicated a higher level of cleanliness and vice versa [11]. Hygiene spot check observation was performed on all the participants by a single team of data collectors to minimize subjectivity.

Preventive health care service use was assessed by the preventive health-seeking index. This index included three variables, i.e., whether the child had been taken to growth monitoring in the previous month and whether the child had received pentavalent and measles immunizations. A score of -1 was given for children who had not received the immunization or had not attended growth monitoring in the previous month, and 0 was given for those who had done so. The index scores ranged from -2 to 0 [33]. As a child becomes eligible for the specific vaccine when it reaches a certain age, the variables related to immunization (i.e., whether the child had received BCG, Pentavalent, OPV and measles) were included in the index for the relevant age groups.

The child was considered ill if s/he had symptoms of either diarrhea or acute respiratory infection (ARI) within 30 days as per maternal recall.

According to BMI, maternal nutritional status was measured. Undernutrition was considered if her BMI was <18.5 kg/m^2^ [34].

Child nutritional status was assessed as per WHO recommended length/height-for-age Z-score (HAZ), weight-for-height Z-score (WHZ) and weight-for-age Z-score (WAZ). The child was defined as stunted, wasted or underweight if their HAZ, WHZ or WAZ were less than -2 standard deviations (SD) [35].

### Data analysis

Data analysis began with descriptive analysis. Means and SD were calculated for continuous variables, while frequencies and percentages were calculated for categorical variables. HAZ, WHZ and WAZ were calculated from the child’s age, height and weight using software named ‘WHO Anthro’ [36]. Bivariate analysis was performed to determine the factors associated with undernutrition. The variables of interest in the bivariate analysis were the child’s age and sex, maternal age, education occupation and nutritional status, SES, household food security, monthly family income, family type, family size, the number of children under five years of age in the household and maternal mental health. Bivariate analysis was also performed to determine the association between maternal mental health and childcare practices and between childcare practices and child nutritional status. Chi-squared test, Fisher’s exact test, Mann-Whitney U test and Spearman correlation were carried out as appropriate. A chi-squared test was performed to determine the association between maternal mental health and child nutritional status. Multivariable logistic regression was conducted after controlling for the other factors (background variables of the sample) that showed a significant association in bivariate analyses. Statistical significance was defined as *p*<0.05. Data analysis was performed using IBM SPSS version 21.0.

### Ethical approval

Ethical approval was obtained from the ethical committee of the National Institute of Preventive and Social Medicine (NIPSOM), Bangladesh. The approval number of this study is 2013/1391. Informed written consent was taken from the mothers. They were informed about the study aims, procedure, benefits or harm of participation, right to participate or refuse and confidentiality of information.

## Results

### Background characteristics

The background characteristics of the respondents are described in Table 1. Infants were less common (14.0%) than other age groups, whereas the age group of 24–35 months consisted of a maximum number of children (31.4%). There were more females than males, with a male-female ratio of 1:1.2. The age of the mothers ranged from 15 to 44 years, with a mean of 25.30±5.65 years. A higher proportion of mothers were educated up to the primary level (40.9%), were housewives (89.8%) and belonged to low socioeconomic status (64.4). More than half (52.7%) of the respondents belonged to a moderate food insecure state, and only one-tenth (12.9%) were in a food secure state. The monthly family income ranged from 2,000 BDT to 50,000 BDT, with an average of 10595 ± 5234 BDT. Most of the respondents belonged to a nuclear family (83%). The total family member ranged from 3 to 12, and the family size of the maximum respondents was small (59.5%). Out of 264 mothers, 46.2% suffered from CMD. The prevalence of child stunting, wasting and underweight were 44.3%, 18.2% and 33.7%, respectively.

**Table 1:** Background characteristics of the sample.

Background characteristics	N (%)
**Age of the children in months**	
<6	25 (9.5)
6–11	12 (4.5)
12–23	68 (25.8)
24–35	83 (31.4)
36–59	76 (28.8)
**Sex of the children**	
Male	122 (46.2)
Female	142 (53.8)
**Age of the mothers in years**	
<20	30 (11.4)
20–24	101 (38.3)
25–29	66 (25.0)
30–34	39 (14.8)
≥35	28 (10.5)
Mean ± SD	25.30 ± 5.65
**Educational status of the mothers**	
Illiterate	72 (27.3)
Primary	108 (40.9)
Class 6–8	56 (21.2)
Class 9–10	26 (9.8)
Class 11–12	2 (0.8)
**Occupation of the mothers**	
Housewife	237 (89.8)
Working	27 (10.2)
**Underweight status of mother**	
No	207 (78.4)
Yes	57 (21.6)
**Socioeconomic class**	
Lower	170 (64.4)
Lower middle	75 (28.4)
Upper middle	19 (7.2)
**Household food security**	
Food secure	34 (12.9)
Mildly food insecure access	63 (23.9)
Moderately food insecure access	139 (52.7)
Severely food insecure access	28 (10.6)
**Monthly family income in BDT**	
Up to 6500	51 (19.3)
6501–13000	157 (59.5)
13000 and above	56 (21.2)
Mean ± SD	10595 ± 5234
**Family type**	
Single	219 (83.0)
Extended	45 (17.0)
**Family size**	
Small	157 (59.5)
Medium	78 (29.5)
Large	29 (11.0)
**Number of under-five children**	
One child	230 (87.1)
More than one	34 (12.9)
**Maternal mental health**	
No maternal CMD	142 (53.8)
Maternal CMD	122 (46.2)
**Child nutritional status**	
Stunting	117 (44.3)
Wasting	48 (18.2)
Underweight	89 (33.7)

### Maternal mental health, childcare practice and child illness

The associations of maternal mental health with child feeding practice, hygiene practice, preventive care service use and child illness are shown in S1 Table. Out of 264 children, good, average and poor feeding practice was 25.8%, 48.5% and 25.7%, respectively. Poor feeding practices were more than three times higher among mothers with CMD (41.8%) than those with no CMD (12.0%). The mean child’s hygiene score was lower (2.57 ± 1.02) in the mothers with CMD than those with no CMD (3.42 ± 0.85). Similarly, the mothers’ hygiene score was lower in the mothers with CMD (3.30±0.79) than those with no CMD (3.77 ± 0.55). The mean preventive care score was less (-1.24 ± 0.53) among the mothers whose mental health was poor than whose mental health was normal (-1.08 ± 0.50). The diarrheal disease was more common among the children of the mothers with CMD (54.4%) than normal mothers (38.6%).

### Childcare practice and nutritional status

The associations of child feeding practices and child illness with child nutritional status are illustrated in S2 Table. Child stunting was less with high feeding practice (35.3%) and higher with low feeding practice (54.4%). The proportion of wasting was two times higher among children with medium feeding practice (18.0%) and four times higher among the children with low feeding practice (29.2%) than the children with high feeding practice (7.4%). Child underweight was about three times and five times more common among the children with medium (35.9%) and low feeding practice (52.9%), respectively than the children with high feeding practice (10.3%). Both HAZ and WAZ scores increased with the child’s hygiene and mother’s hygiene scores. There was no relationship between preventive care service use and HAZ, WHZ and WAZ. The proportion of stunting, wasting, or underweight was more common in the children who had a history of diarrhea within 30 days than in those who did not.

### Sociodemographic characteristics and child nutritional status

The associations of child characteristics, maternal characteristics and household characteristics with HAZ, WHZ and WAZ are shown in the S3 Table. Child age and sex were associated with child stunting. Stunting was less common in male children (26.2%) than in female ones (59.9%). The percentage of stunted children was highest (54.4%) in the 12–23 months age group and lowest (12%) among those under six months. The factors associated with wasting were child sex, the number of under-five children in the household and maternal mental health. The percentage of wasting was higher in female children (25.4%) than in male children (9.8%). Wasting was more common in families with more than one child (38.2%) than in those with a single child (15.5%). The proportion of wasted children was higher among mothers with CMD (25.4%) than mothers with no CMD (12.0%). The factors contributing to child underweight were child sex, maternal nutrition, household food insecurity and maternal mental health. The percentage of wasting was about three times higher in female children (25.4%) than in male children (9.8%). Child underweight was more common in case of underweight mothers (52.6%) than the normal mothers (28.5%). The proportion of underweight children increased with the increased level of food insecurity. It was highest in severely food insecure households (50.0%) and lowest in food-secure households (14.7%). Underweight children were more common among mothers with CMD (45.1%) than mothers with no CMD (23.9%).

### Maternal mental health and child nutritional status

Child stunting was more common in the mothers with CMD (50.0%) than in the mothers with no CMD (39.4%). This difference was not statistically significant (*p =* 0.085, Table 2).

**Table 2:** Association between maternal mental health and child nutritional status.

Maternal mental health	Height-for age Z-score		Weight-for-height Z-score		Weight-for-age Z-score	
	Normal	Stunting	Normal	Wasting	Normal	Underweight
	(n = 147)	(n = 117)	(n = 216)	(n = 48)	(n = 175)	(n = 89)
	N (%)	N (%)	N (%)	N (%)	N (%)	N (%)
**No CMD** ^a^	86 (60.6)	56 (39.4)	125 (88.0)	17 (12.0)	108 (76.1)	34 (23.9)
**CMD**	61 (50.0)	61 (50.0)	91 (74.6)	31 (25.4)	67 (54.9)	55 (45.1)
**Crude OR (95% CI)**	1.54 (0.94–2.51)		**2.51 (1.31–4.80)**		**2.61 (1.54–4.41)**	
***p-*value**	0.085		**0.005**		**<0.001**	
**Adjusted OR (95% CI)**	1.46 (0.84–2.54)^b^		**2.25 (1.15–4.43)** ^c^		1.77 (0.94–3.33)^d^	

^a^Reference

^b^Adjusted for maternal age, child age and sex

^c^Adjusted for child sex and number of under-5 children in the household

^d^Adjusted for maternal nutrition, child sex and household food security

Approximately one-fourth of the mothers (25.4%) with CMD had wasted children, whereas only 12% of the mothers with no CMD had wasted children, which was statistically significant (*p =* 0.005). The logistic regression model contained three independent variables (maternal mental health, child sex and the number of under-five children in the household). The final model was statistically significant (*p<*0.001). The mothers with CMD were approximately two times more likely to have wasted children than the mothers with no CMD after adjusting for the effect of child sex and number of children under five in the household (aOR = 2.25, 95% CI = 1.15–4.43, Table 2).

Underweight children were more common in mothers with CMD (45.1%) than in mothers with no CMD (23.9%). This difference was statistically significant (*p* < 0.001). The logistic regression model contained four independent variables (maternal mental health, maternal nutritional status, child sex and household food security). The final model was statistically significant (*p<*0.001). The association between maternal CMD and child underweight was attenuated and no longer statistically significant after controlling for the effect of maternal nutrition, child sex and household food security (aOR = 1.77, 95% CI = 0.94–3.33, Table 2).

## Discussion

This study revealed maternal CMD as a considerable public health problem associated with child undernutrition. Approximately half of the mothers (46.2%) were identified as suffering from CMD in this study. This result is similar (49%) to a previous study carried out in Bangladesh [11]. Although maternal CMD is very common in developing countries, its prevalence differs from country to country: Vietnam 31.2%; Ethiopia 39.4% [11]; Peru 30% and India 30.0% [10]. The high prevalence of maternal CMD in developing countries might be due to lower socioeconomic conditions, food insecurity, being younger, being illiterate, undernutrition, unsupportive partners and experiencing physical violence [11, 37].

Evidence of an association between maternal CMD and poor nutritional status in the urban population of Bangladesh in this study confirmed previous evidence from other countries [10, 11, 19, 28]. After controlling for the effects of the confounders, a significant association was found between maternal CMD and child wasting. An association was found with underweight in the bivariate analysis, but it was attenuated and no longer statistically significant in the multivariable analysis suggesting that other factors might be more important or so tightly correlated with maternal mental health that the independent effects cannot be disentangled in this model. Surprisingly, there was no statistically significant association between maternal CMD and stunting, which is hard to explain. Stunting is a condition that reflects the cumulative effects of chronic malnutrition, while wasting is a condition that reflects acute or recent nutritional deficits. Underweight reflects the combination of acute and chronic malnutrition. The significant association between maternal CMD and child wasting might be bidirectional. As wasting is an acute condition, it could also affect maternal mental health. The SRQ-20 used to assess maternal mental health, is based on some symptoms in the previous 30 days. Therefore, the association of maternal CMD with wasting, which was found in this study, is justifiable. However, this result is inconsistent with Harpham et al. and Nguyen et al. Maternal CMD is associated with child stunting in India and child underweight in Vietnam [10]. A study conducted in Bangladesh found that maternal CMD was associated with stunting but no association with wasting or underweight [11]. The variability of findings may be explained by sociocultural differences in care and feeding practices, differences in maternal education, socioeconomic status, household food security and other factors. This heterogeneity in the results is particularly interesting and needs to be further analyzed to understand this relationship.

Childcare practices are identified as a key underlying cause of child malnutrition. The mental health of caregivers is one of the components that affect child care practice, as stated in the UNICEF care model [38]. Several studies have revealed that maternal mental health adversely affects child feeding practices resulting in malnutrition [10, 11]. In the present study, childcare practices were measured in three domains–feeding practice, hygiene practice and preventive care service use. Maternal mental health was found to be associated with all three domains. Maternal CMD could prevent them from taking proper care of their children, especially infant feeding, improper food preparation and child care regarding immunization. Therefore, it constitutes a risk factor for impairment of the nutritional status of the children [16, 20].

This study found an association of child feeding practices with wasting and underweight. A study conducted in Latin America also found an association between child feeding practices and nutritional status [39]. The important factors of hygiene practice are social, lifestyle, and environmental factors, which are indirectly associated with the mental health of family members [40]. A significant relationship was observed between hygiene practice and child stunting and wasting. A similar finding was observed in our analysis of the relationship between individual hygiene and child nutrition [39]. This might be because inadequate hygiene practices increase the risk of infection among children, especially diarrhea, helminth infection and other gastrointestinal symptoms [41]. Harpham et al. have also described a pathway showing how maternal CMD affects child nutrition through breastfeeding, child immunization and child physical health [10].

The association between maternal CMD and child illness was examined in this study. The diarrheal disease was significantly associated with maternal CMD. To the best of our knowledge, few studies have examined the association between maternal mental health and child illness [11, 42, 43]. The association between maternal CMD and diarrhea can be explained by poor maternal mental health affecting the ability of mothers to take on their personal hygiene, which translates into diarrheal diseases among children. These findings suggest that a potential pathway for the relationship between poor maternal mental health and child undernutrition could be through child illness. Poor mental health may hinder the mother’s ability to take adequate care of her child, prevent illness and seek health care when the child is ill.

Despite all efforts, there were some limitations in this study. First, the study was conducted in a selected urban area. Therefore, the study result might not necessarily have external validity. Second, the SRQ-20 is a screening tool to assess mental health, not a diagnostic tool. Therefore, the number of mothers identified as having CMD might differ from the actual number. Hygiene spot check observation might be biased. Third, data were collected based on maternal recall of their mental distress in the past four weeks prior to the interview. Maternal recall can result in either under- or over-reporting. Fourth, the association of childcare practice with maternal mental health and child nutritional status was investigated by bivariate analysis. The logistic regression model was not used to address this. Fifth, as it was a cross-sectional study, a causal relationship between maternal mental health and child nutrition was not established. The potential reverse causality cannot be ruled out as poor child health is a contributor to maternal CMD, and this may be especially true when children are acutely undernourished rather than chronically moderately undernourished. This, in turn, may impede the ability of the mother to take adequate care of the child. Last, data for this study were collected about eight years ago (in 2013), which poses serious questions considering the factors that might have affected the generalizability of the findings in this clime and the effect of the COVID-19 pandemic on maternal mental health and child nutritional status.

The study results may contribute to understanding the importance of maternal mental health as a public health problem and its potential pathways to cause child malnutrition. It is time to invest in fully understanding risk factors for CMD and develop evidence-based mental health interventions to improve maternal mental health. Further research with a structural equation modelling approach may provide more information to fully comprehend the complexities of the relationship between maternal mental health and child nutrition status. Interventions to address maternal mental health in child nutrition programs might contribute to improving nutritional status of the children.

## Supporting information

S1 Fig Conceptual framework. (TIF)

S1 Table Association of maternal mental health with childcare practices and child illness. (DOCX)

S2 Table Association of childcare practice and child illness with child nutritional status. (DOCX)

S3 Table Association between background characteristics of the sample and child nutrition. (DOCX)

S1 Dataset (SAV)
