# Supplementary figures and images for: Maternal mental health and child nutritional status in an urban slum in Bangladesh: A cross-sectional study
Source: PLOS Glob Public Health. 2022 Oct 19;2(10):e0000871. doi: 10.1371/journal.pgph.0000871 (PMC10021263; doi:10.1371/journal.pgph.0000871)

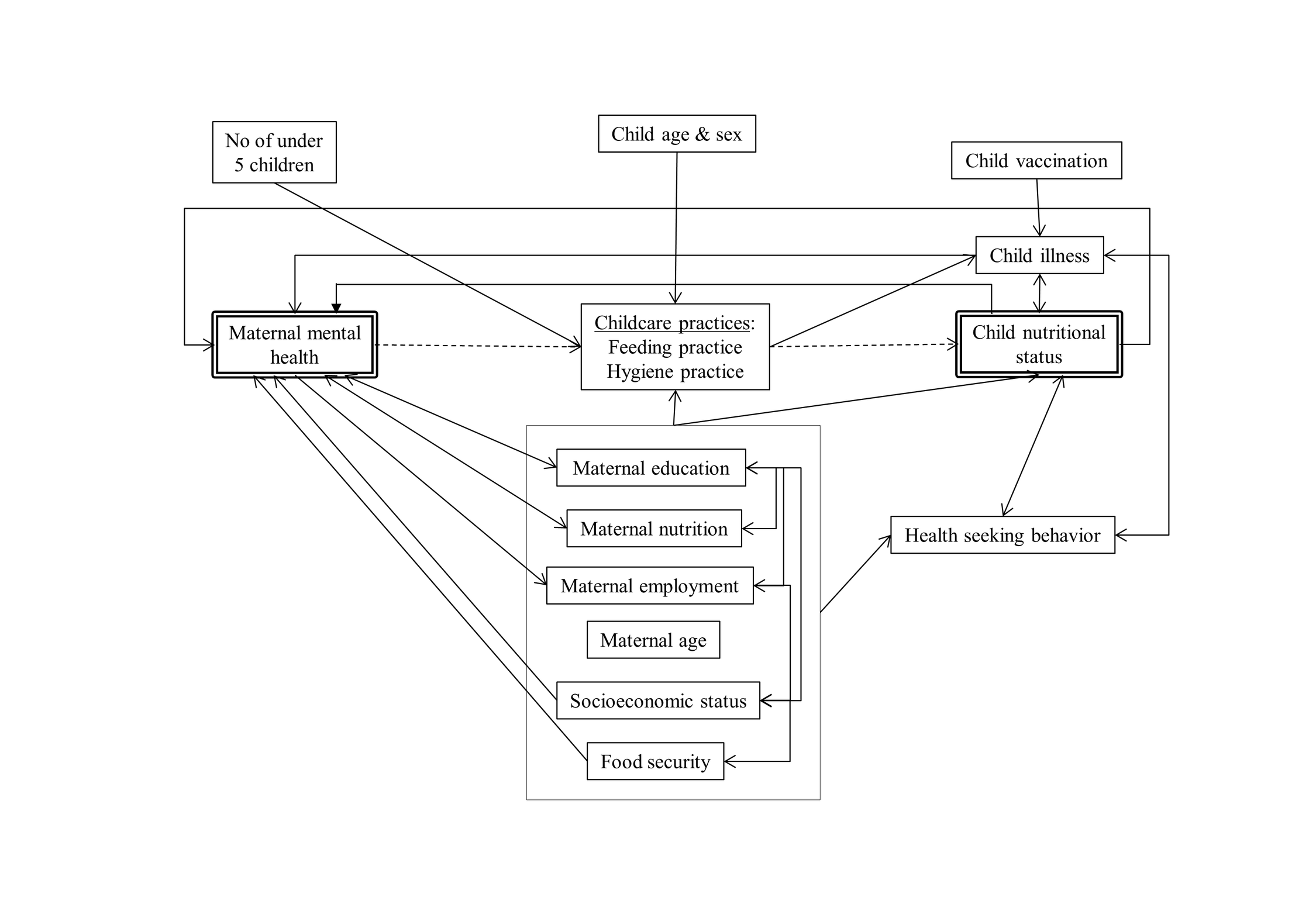

Supplement: S1 Fig — (TIF) [file pgph.0000871.s001.tif]
