# Supplementary material for: Maternal mental health and child nutritional status in an urban slum in Bangladesh: A cross-sectional study
Source: PLOS Glob Public Health. 2022 Oct 19;2(10):e0000871. doi: 10.1371/journal.pgph.0000871 (PMC10021263; doi:10.1371/journal.pgph.0000871)
Supplement: S1 Table — (DOCX) [file pgph.0000871.s002.docx]

**S1 Table. Association of maternal mental health with childcare practices and child illness**

| **Childcare practices** | **No maternal CMD**  **n=142** | **Maternal CMD**  **n=122** | ***p*-value** |
| --- | --- | --- | --- |
| **Child feeding practice** | **N (%)** | **N (%)** |  |
| Good | 59 (41.5) | 9 (7.4) | **<0.001**^a^ |
| Average | 66 (46.5) | 62 (50.8) |  |
| Poor | 17 (12.0) | 51 (41.8) |  |
| **Hygiene practice** |  |  |  |
| Child hygiene score (Mean±SD) | 3.42 ± 0.85 | 2.57 ± 1.02 | **<0.001**^b^ |
| Mother’s hygiene score (Mean±SD) | 3.77 ± 0.55 | 3.30 ± 0.79 | **<0.001**^b^ |
| **Preventive care service use** |  |  |  |
| Preventive health seeking index (Mean±SD) | -1.08 ± 0.50 | -1.24 ± 0.53 | **0.016**^b^ |
| **Child illness** |  |  |  |
| Diarrhea | 22 (38.6) | 43 (54.4) | 0.049^a^ |
| ARI | 4 (7.1) | 9 (11.4) | 0.410^a^ |

^a^ Chi-Squared test; ^b^ Mann-Whitney U Test
