# Supplementary material for: Maternal mental health and child nutritional status in an urban slum in Bangladesh: A cross-sectional study
Source: PLOS Glob Public Health. 2022 Oct 19;2(10):e0000871. doi: 10.1371/journal.pgph.0000871 (PMC10021263; doi:10.1371/journal.pgph.0000871)
Supplement: S2 Table — (DOCX) [file pgph.0000871.s003.docx]

**S2 Table. Association of childcare practice and child illness with child nutritional status**

| **Childcare practice** | **Height-for age Z-score** | | | **Weight-for-height Z-score** | | | **Weight-for-age Z-score** | |  |
| --- | --- | --- | --- | --- | --- | --- | --- | --- | --- |
|  | **Normal**  **(n=147)** | **Stunting**  **(n=117)** | ***p-value*** | **Normal**  **(n=216)** | **Wasting**  **(n=48)** | ***p-value*** | **Normal**  **(n=175)** | **Underweight**  **(n=89)** | ***p-value*** |
|  | **N (%)** | **N (%)** |  | **N (%)** | **N (%)** |  | **N (%)** | **N (%)** |  |
| **Child feeding practice** | | | | | | | | | |
| **Good** | 44 (64.7) | 24 (35.3) |  | 63 (92.6) | 5 (7.4) |  | 61 (89.7) | 7 (10.3) |  |
| **Average** | 72 (56.2) | 56 (43.8) | 0.079 ^a^ | 105 (82.0) | 23 (18.0) | **0.004** ^a^ | 82 (64.1) | 46 (35.9) | <**0.001**^a^ |
| **Poor** | 31 (45.6) | 37 (54.4) |  | 48 (70.6) | 20 (29.2) |  | 32 (47.1) | 36 (52.9) |  |
| **Child’s hygiene practice** | rho = 0.158 | | **0.010** ^b^ | rho = 0.102 | | 0.098 ^b^ | rho = 0.167 | | **0.006**^b^ |
| **Mother’s hygiene practice** | rho = 0.132 | | **0.032** ^b^ | rho = 0.097 | | 0.117 ^b^ | rho = 0.124 | | **0.045**^b^ |
| **Preventive care service use** | rho = -0.026 | | 0.673 ^b^ | rho = -0.017 | | 0.781 ^b^ | rho = -0.042 | | 0.496^b^ |
| **Diarrhea** | | | | | | | | | |
| **No** | 48 (67.6) | 23 (32.4) | **0.019^a^** | 59 (83.1) | 12 (16.9) | 0.266^a^ | 51 (71.8) | 20 (28.8) | **0.046^a^** |
| **Yes** | 31 (47.7) | 34 (52.3) |  | 49 (75.4) | 16 (24.6) |  | 36 (55.5) | 29 (44.6) |  |
| **ARI** | | | | | | | | | |
| **No** | 72 (59.0) | 50 (41.0) | 0.797^a^ | 99 (81.1) | 23 (18.9) | 0.097^c^ | 79 (64.8) | 43 (35.2) | 0.437^a^ |
| **Yes** | 6 (46.2) | 7 (53.8) |  | 8 (61.5) | 5 (38.5) |  | 7 (53.8) | 6 (46.2) |  |

^a^Chi-Squared test; ^b^Spearman correlation; ^c^Fisher’s Exact Test
