# Supplementary material for: Maternal mental health and child nutritional status in an urban slum in Bangladesh: A cross-sectional study
Source: PLOS Glob Public Health. 2022 Oct 19;2(10):e0000871. doi: 10.1371/journal.pgph.0000871 (PMC10021263; doi:10.1371/journal.pgph.0000871)
Supplement: S3 Table — (DOCX) [file pgph.0000871.s004.docx]

**S3 Table.** **Association between background characteristics of the sample and child nutrition**

| **Characteristics** | **Height-for age Z-score** | | | **Weight-for-height Z-score** | | | **Weight-for-age Z-score** | | |
| --- | --- | --- | --- | --- | --- | --- | --- | --- | --- |
|  | **Normal**  **(n=147)** | **Stunting**  **(n=117)** | ***p-value ^a^*** | **Normal (n=216)** | **Wasting**  **(n=48)** | ***p-value ^a^*** | **Normal (n=175)** | **Under weight (n=89)** | ***p-value ^a^*** |
|  | **N (%)** | **N (%)** |  | **N (%)** | **N (%)** |  | **N (%)** | **N (%)** |  |
| **Age of the mother in years** | | | | | | | | | |
| <20 | 16 (53.3) | 14 (46.7) | 0.490 | 24 (80.0) | 6 (20.0) | 0.284 | 19 (63.3) | 11 (36.7) | 0.369 |
| 20-24 | 50 (49.5) | 51 (50.5) |  | 82 (82.2) | 19 (18.8) |  | 62 (61.4) | 39 (38.6) |  |
| 25-29 | 40 (60.6) | 26 (39.4) |  | 51 (77.3) | 15 (22.7) |  | 47 (71.2) | 19 (28.8) |  |
| 30-34 | 25 (64.1) | 14 (35.9) |  | 32 (82.1) | 7 (17.9) |  | 30 (76.9) | 9 (23.1) |  |
| ≥35 | 16 (57.1) | 12 (42.9) |  | 27 (96.4) | 1 (3.6) |  | 17 (60.7) | 11 (39.3) |  |
| **Educational status of mother** | | | | | | | | | |
| Illiterate | 42 (58.3) | 30 (41.7) | 0.890 | 61 (84.7) | 11 (15.3) | 0.111 | 49 (68.1) | 23 (31.9) | 0.970 |
| Primary | 61 (56.5) | 47 (43.5) |  | 84 (77.8) | 24 (22.2) |  | 71 (65.7) | 37 (34.3) |  |
| Class 6-8 | 29 (51.8) | 27 (48.2) |  | 44 (78.6) | 12 (21.4) |  | 36 (64.3) | 20 (35.7) |  |
| ≥Class 9 | 15 (53.6) | 13 (46.4) |  | 27 (96.4) | 1 (3.6) |  | 19 (67.9) | 9 (32.1) |  |
| **Occupation of mother** | | | | | | | | | |
| Housewife | 134 (56.5) | 103 (43.5) | 0.406 | 191 (80.6) | 46 (19.4) | 0.126 | 159 (67.1) | 78 (32.9) | 0.415 |
| Working | 13 (48.1) | 14 (51.9) |  | 25 (92.6) | 2 (7.4) |  | 16 (59.3) | 11 (40.7) |  |
| **Underweight status of mother** | | | | | | | | | |
| No | 121 (58.5) | 86 (41.5) | 0.084 | 174 (84.1) | 33 (15.9) | 0.072 | 148 (71.5) | 59 (28.5) | **0.001** |
| Yes | 26 (45.6) | 31 (54.4) |  | 42 (73.7) | 15 (26.3) |  | 27 (47.4) | 30 (52.6) |  |
| **Child sex** | | | | | | | | | |
| Male | 90 (73.8) | 32 (26.2) | **<0.001** | 110 (90.2) | 12 (9.8) | **0.001** | 105 (86.1) | 17 (13.9) | **<0.001** |
| Female | 57 (40.1) | 85 (59.9) |  | 106 (74.6) | 36 (25.4) |  | 70 (49.3) | 72 (50.7) |  |
| **Age of the children in months** | | | | | | | | | |
| <6 | 22 (88.0) | 3 (12.0) | **0.002** | 18 (72.0) | 7 (28.0) | 0.173^b^ | 21 (84.0) | 4 (16.0) | 0.290 |
| 6-11 | 8 (66.7) | 4 (33.3) |  | 8 (66.7) | 4 (33.3) |  | 8 (66.7) | 4 (33.3) |  |
| 12-23 | 31 (45.6) | 37 (54.4) |  | 54 (79.4) | 14 (20.6) |  | 41 (60.3) | 27 (39.7) |  |
| 24-35 | 40 (48.2) | 43 (51.8) |  | 73 (88.0) | 10 (12.0) |  | 53 (63.9) | 30 (36.1) |  |
| 36-59 | 46 (60.5) | 30 (39.5) |  | 63 (82.9) | 13 (17.1) |  | 52 (68.4) | 24 (31.6) |  |
| **Socioeconomic status** | | | | | | | | | |
| Lower | 92 (54.1) | 78 (45.9) | 0.711 | 140 (82.4) | 30 (17.6) | 0.527 | 109 (64.1) | 61 (35.9) | 0.403 |
| Lower middle | 43 (57.3) | 32 (42.7) |  | 59 (78.7) | 16 (21.3) |  | 51 (68.0) | 24 (32.0) |  |
| Upper middle | 12 (63.2) | 7 (36.8) |  | 17 (89.5) | 2 (10.5) |  | 15 (78.9) | 4 (21.1) |  |
| **Household food security** | | | | | | | | | |
| Food secure | 22 (64.7) | 12 (35.3) | 0.635 | 31 (91.2) | 3 (8.8) | 0.113 | 29 (85.3) | 5 (14.7) | **0.001** |
| Mildly food insecure | 36 (57.1) | 27 (42.9) |  | 55 (87.3) | 8 (12.7) |  | 50 (79.4) | 13 (20.6) |  |
| Moderately food insecure | 75 (54.0) | 64 (46.0) |  | 110 (79.1) | 29 (20.9) |  | 82 (59.0) | 57 (41.0) |  |
| Severely food insecure | 14 (50.0) | 14 (50.0) |  | 20 (71.4) | 8 (28.6) |  | 14 (50.0) | 14 (50.0) |  |
| **Family type** | | | | | | | | | |
| Nuclear family | 122 (55.7) | 97 (44.3) | 0.985 | 181 (82.6) | 38 (17.4) | 0.595 | 146 (66.7) | 73 (33.3) | 0.774 |
| Joint family | 25 (55.6) | 20 (44.4) |  | 35 (77.8) | 10 (22.2) |  | 29 (64.4) | 16 (35.6) |  |
| **Family size** | | | | | | | | | |
| Small | 85 (54.1) | 72 (45.9) | 0.827 | 133 (84.7) | 24 (15.3) | 0.323 | 103 (65.6) | 54 (34.4) | 0.754 |
| Medium | 45 (57.7) | 33 (42.3) |  | 61 (78.2) | 17 (21.8) |  | 54 (69.2) | 24 (30.8) |  |
| Large | 17 (58.6) | 12 (41.4) |  | 22 (75.9) | 7 (24.1) |  | 18 (62.1) | 11 (37.9) |  |
| **No. of under five children** | | | | | | | | | |
| One child | 133 (57.8) | 97 (42.2) | 0.068 | 195 (84.8) | 35 (15.2) | **0.001** | 157 (68.3) | 73 (31.7) | 0.078 |
| More than one | 14 (42.2) | 20 (58.8) |  | 21 (61.8) | 13 (38.2) |  | 18 (52.9) | 16 (47.1) |  |
| **Maternal mental health** | | | | | | | | | |
| No CMD | 86 (60.6) | 56 (39.4) | 0.085 | 125 (88.0) | 17 (12.0) | **0.005** | 108 (76.1) | 34 (23.9) | **<0.001** |
| CMD | 61 (50.0) | 61 (50.0) |  | 91 (74.6) | 31 (25.4) |  | 67 (54.9) | 55 (45.1) |  |

^a^ Chi-Squared test; ^b^ Fisher’s Exact Test
